# Establishing an Efficient Genetic Manipulation System for Sulfated Echinocandin Producing Fungus *Coleophoma empetri*

**DOI:** 10.3389/fmicb.2021.734780

**Published:** 2021-08-20

**Authors:** Ping Men, Min Wang, Jinda Li, Ce Geng, Xuenian Huang, Xuefeng Lu

**Affiliations:** ^1^Shandong Provincial Key Laboratory of Synthetic Biology, Qingdao Institute of Bioenergy and Bioprocess Technology, Chinese Academy of Sciences, Qingdao, China; ^2^Shandong Energy Institute, Qingdao, China; ^3^Qingdao New Energy Shandong Laboratory, Qingdao, China; ^4^College of Life Science, University of Chinese Academy of Sciences, Beijing, China; ^5^Marine Biology and Biotechnology Laboratory, Qingdao National Laboratory for Marine Science and Technology, Qingdao, China; ^6^Key Laboratory of Biofuels, Qingdao Institute of Bioenergy and Bioprocess Technology, Chinese Academy of Sciences, Qingdao, China

**Keywords:** protoplast transformation, promoter, marker, nonhomologous end-joining, *Coleophoma empetri*, melanin biosynthesis, micafungin

## Abstract

Micafungin is an important echinocandin antifungal agent for the treatment of invasive fungal infections. In industry, micafungin is derived from the natural product FR901379, which is a non-ribosomal cyclic hexapeptide produced by the filamentous fungus *Coleophoma empetri*. The difficulty of genetic manipulation in *C. empetri* restricts the clarification of FR901379 biosynthetic mechanism. In this work, we developed an efficient genetic manipulation system in the industrial FR901379-producing strain *C. empetri* MEFC009. Firstly, a convenient protoplast-mediated transformation (PMT) method was developed. Secondly, with this transformation method, the essential genetic elements were verified. Selectable markers *hph*, *neo*, and *nat* can be used for the transformation, and promotors *Ppgk*, *PgpdA*, and *PgpdAt* are functional in *C. empetri* MEFC009. Thirdly, the frequency of homologous recombination was improved from 4 to 100% by deleting the *ku80* gene, resulting in an excellent chassis cell for gene-targeting. Additionally, the advantage of this genetic manipulation system was demonstrated in the identification of the polyketide synthase (PKS) responsible for the biosynthesis of dihydroxynapthalene (DHN)-melanin. This genetic manipulation system will be a useful platform for the research of FR901379 and further genome mining of secondary metabolites in *C. empetri*.

## Introduction

Echinocandins are a family of cyclic lipohexapeptides with the excellent activity of anti-*Candida* and anti-*Aspergillus*, which are considered to be the most potential antifungal agents clinically (Denning, 2003; Patil and Majumdar, 2017). To date, three semisynthetic echinocandins, caspofungin, micafungin, and anidulafungin have been approved for the treatment of invasive fungal infections (IFIs). Among them, micafungin is the only sulfated echinocandin agent (Hashimoto, 2009). The biosynthetic pathway of the lead compounds of caspofungin and anidulafungin have been identified in 2012 and 2013, respectively (Cacho et al., 2012; Chen et al., 2013; Hüttel, 2017). Compound FR901379 produced by filamentous fungi *Coleophoma empetri* is the lead compound of micafungin. The biosynthesis pathway of FR901379 is still cryptic, because of the difficulty of genetic manipulation of *C. empetri*. In 2009, Masato Yamada developed *Agrobacterium tumefaciens*-mediated transformation method (ATMT) in the *C. empetri* F11899, only the fragment of hygromycin B resistant gene was successfully integrated into the genome by random insertion (Yamada et al., 2009). More efficient and systematic genetic engineering tools would be conducive to functional genomics studies and are also fundamental to the metabolic engineering of industrial strains. Therefore, it is still necessary to explore more effective transformation methods and available genetic elements.

Several fundamental points must be considered to construct an efficient genetic manipulation system in filamentous fungi. The primary one is a convenient and efficient genetic transformation method. The protoplast-mediated transformation (PMT) and ATMT are the two most commonly used genetic transformation methods in filamentous fungi (Meyer, 2008). Among them, PMT method is usually more preferred, because it is not necessary to construct a binary vector and the corresponding *A. tumefaciens* strain (Michielse et al., 2008). The second is an efficient gene-targeting system. Disrupting the nonhomologous end-joining (NHEJ) pathways is a general way to improve the frequency of gene-targeting in filamentous fungi (Ninomiya et al., 2004). Even in the CRISPR/Cas9 genome editing system, the chassis cell of NHEJ deficient is also inevitable (Wei et al., 2020). In addition, the elements of promoter and selectable marker are also indispensable, especially for the sequential metabolic engineering of industrial strains (Blumhoff et al., 2013).

In this work, a systematic and useful genetic manipulation system was established in an excellent FR901379 producing strain *C. empetri* MEFC009. It includes four key points: efficient protoplast-mediated transformation method, available promoters (*PgpdA*, *PgpdAt*, and *Ppgk*), confirmed selectable markers (*hph*, *neo*, and *nat*), and *ku80* deficient chassis cell with high homologous recombination frequency. Furthermore, the polyketide synthase responsible for the biosynthesis of melanin was efficiently identified using this genetic manipulation system. This genetic manipulation system will be significantly conducive to the study of FR901379 biosynthetic pathway and further sequential metabolic engineering.

## Materials and Methods

### Chemicals and Reagents

Antibiotics were purchased from Solarbio (Beijing China). calcofluor white (CFW) was obtained from Sigma-Aldrich (United States), 4',6-diamidino-2-phenylindole (DAPI) was obtained from Beyotime (Shanghai, China). Lysis enzymes are obtained from Sigma (United States), snailase is obtained from Solarbio (Beijing China), and lywallzyme is obtained from Guangdong Microbial Culture Collection Center (Guangdong, China).

### Strains, Medium, and Cultivation Conditions

All strains used in this study are listed in Table 1. The *C. empetri* MEFC009 and all mutant strains were grown on PDA. Cultivation in shake flasks was carried out in seed medium (MKS: soluble starch 15g/L, sucrose 10g/L, cotton seed meal 5g/L, peptone 10g/L, KH_2_PO_4_ 1g/L, CaCO_3_ 2g/L, and pH 6.5) and FR901379 production medium (MKF: glucose 10g/L, corn starch 30g/L, peptone 10g/L, (NH_4_)_2_SO_4_ 6g/L, KH_2_PO_4_ 1g/L, FeSO_4_ 0.3g/L, ZnSO_4_ 0.01g/L, CaCO_3_ 2g/L, and pH 6.5).

**Table 1:** *C. empetri* MEFC009 strains used in this study.

Strain	Genotypes	Reference of source
MEFC009	*Coleophoma empetri* MEFC009 wild-type	
MEFC009-hph	Mutant strain of MEFC009 harboring resistance gene *hph*	This study
MEFC009-neo	Mutant strain of MEFC009 harboring resistance gene *neo*	This study
MEFC009-nat	Mutant strain of MEFC009 harboring resistance gene *nat*	This study
MEFC009-*PgpdA*	Mutant strain of MEFC009 harboring *PgpdA-sgfp-hph*	This study
MEFC009-*PgpdAt*	Mutant strain of MEFC009 harboring *PgpdAt-sgfp-hph*	This study
MEFC009-*Ppgk*	Mutant strain of MEFC009 harboring *Ppgk-sgfp-hph*	This study
MEFC009-Δku80-hph	*ku80* disruption mutant of MEFC009 carrying *hph*	This study
MEFC009-Δku80-neo	Mutant strain derived from MEFC009-Δku80-hph carrying *neo*	This study
MEFC009-Δpks11.2	*pks11.2* disruption mutant strain derived from MEFC009-Δku80-neo carrying *hph*	This study

### Staining Nuclei and Cell Wall of the Hyphae

The cell wall and nuclei of the hyphae of *C. empetri* MEFC009 were stained by CFW and DAPI, respectively (Chazotte, 2011). The hyphae of *C. empetri* MEFC009 was immersed in 200μl phosphate buffered saline (PBS pH 7.4, 0.1mol/L), then stained by 0.8μg/ml of DAPI or 0.05μg/ml of CFW for 5min. The hyphae were washed twice by PBS to remove the stain and observed by confocal microscopy (FluoView FV1000). In addition, 0.8μg/ml of DAPI and 0.05μg/ml of CFW were added together to stain the nuclei and cell wall of the hyphae simultaneously. The excitation and emission wavelength of CFW and DAPI is 345/405nm with blue fluorescence.

### Binary Vector Assembly and Transformation of *A. tumefaciens*

All primers used in this study are listed in Supplementary Table S1. The selectable marker gene *nat* was amplified from pPK2natGFPD by PCR using primers PtrpC/nat-R (Gu et al., 2018). The coding DNA sequence (CDS) of *neo* was synthesized fused with promoter *Ppgk* and terminator *Tpgk* by fusion PCR. The markers *nat* and *neo* were cloned into the binary vector pCambia1300 using a One-Step Cloning Kit (Vazyme, Nanjing, China) to construct the recombinant plasmids pPM-3, and pPM-4 (Hršelová et al., 2015). The recombinant *A. tumefaciens* LBA4404 harboring plasmids pCambia1300, pPM-3, and pPM-4 were constructed. The *Agrobacterium*-mediated transformation of *C. empetri* MEFC009 was performed as previously described (Yamada et al., 2009).

### DNA Manipulation for Cassettes Construction

To evaluate the function of promoters *PgpdA*, *Ppgk*, and *PgpdAt*, the cassettes containing the above promoters were constructed, respectively. The DNA cassettes of *PgpdA-sgfp-TtrpC-hph* and *PgpdAt-sgfp-TtrpC-hph* were amplified from plasmid pAN52-4 (Punt et al., 1991) and pXH2-1 (Huang et al., 2014) by PCR using primer pairs PgpdA-F/TtrpC-R and PgpdAt-F/TtrpC-R, respectively. The fragment of *sgfp* of the cassette of *Ppgk-sgfp-Tpgk-hph* was amplified from plasmid pXH2-1, and then fused with *Ppgk*, *Tpgk*, and *hph* by fusion PCR, resulting in cassette *Ppgk-sgfp-Tpgk-hph*.

To knock out the *ku80* gene, the flanking 5' and 3' DNA of the *ku80* gene were amplified by PCR from the genome of *C. empetri* MEFC009 using primer pairs Uku80-F/Uku80-(hph)-R and Dku80-(hph)-F/Dku80-R, and fused with *hph* marker by fusion PCR. The gene-targeting cassette was amplified using primers Uku80-CS-F/Dku80-CS-R. The *ku80* deletion cassette using *neo* marker was constructed through the same approach. The flanking 5' and 3' DNA of *pks11.2* gene were amplified by PCR from the genome of *C. empetri* MEFC009 using primer pairs Upks11.2-F/Upks11.2-R and Dpks11.2-F/Dpks11.2-R and fused with *hph* marker by fusion PCR. The gene-targeting cassette was amplified by primers Upks11.2-CS-F/Dpks11.2-CS-R.

### Protoplast-Mediated Transformation

The transformation mediated by protoplast was developed in *C. empetri* MEFC009 according to the protocol previously described with modification (Marcone et al., 2010). The fresh hyphae from the PDA plate were broken by Superfine homogenizers (Fluko) and cultivated in 50ml of MKS medium for 2days, 220rpm, 25°C. Then hyphae were broken again and inoculated in a new shack flask with 10% proportion, cultivated for 1day at 25°C. The hyphae were harvested by filtration through Miracloth (Calbiochem), washed with 0.6M MgSO_4_, and immersed in the enzymatic solution (0.6% of snailase, 0.6% lysis enzymes, and 0.7% of lywallzyme) for 4h at 30°C to prepare protoplasts. The protoplasts were collected by filtered through Miracloth and centrifuged at 1,500rpm at 4°C for 20min. After washed with 1M D-sorbitol buffer and STC (1M D-sorbitol, 10mM Tris-HCl pH 8.0, 50mM CaCl_2_) successively, the protoplasts were resuspended in STC used for transformation.

To optimize the regeneration medium for protoplasts, the protoplasts suspension was diluted to 10^4^cells/ml with STC and H_2_O, respectively. A 100μl portion of the protoplast dilution was mixed with the regeneration medium and pour into PDAS plates with different concentrations of D-sorbitol: 0, 0.4, 0.6, 0.8, and 1.2M.

As for transformation, approximately 1μg of DNA fragments were mixed with 100μl protoplast solution (1.5×10^7^ protoplasts/ml) and 50μl of ice-cold PSTC (40% PEG 4000, 1M D-sorbitol, 10mM Tris-HCl pH 8.0, and 50mM CaCl_2_), incubating on ice for 25min. Then 1ml of PSTC was added and incubated for 20min at room temperature. Thereafter, the protoplasts mixture was mixed with 20ml of liquid top agar (PDB with 0.5% agarose and 0.8M D-sorbitol), spread on PDAS amended with the antibiotic, and cultured for 5–7days at 30°C.

### Fermentation and HPLC Measurement

*C. empetri* MEFC009 and mutant *C. empetri* MEFC009-Δku80 were inoculated in 50ml of MKF medium at 25°C for 10days. Then 1ml of fermentation culture was taken every 2days and extracted with equal volumes of methanol by ultrasonic crushing for 1h. Four independents were set for each experiment. The amount of FR901379 was determined by high performance liquid chromatography (HPLC) monitored at 210nm. The separation was carried out on a C_18_ reversed phase column (Agilent ZORBAX SB-C18 column, 4.6×150mm, 5μm) at a flow rate of 1ml/min at 30°C, using acetonitrile (ACN)/H_2_O as elution solvents. A linear gradient from 5 to 100% ACN in H_2_O containing 0.1% (v/v) TFA in 20min was used.

## Results and Discussion

### Morphology of Vegetative Hyphae

**Figure 1:**
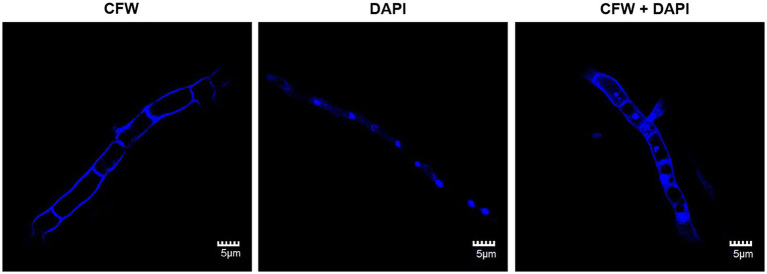
Fluorescence microscopy images of the stained vegetative hyphae of *C. empetri* MEFC009. CFW: CFW-stained image of hyphae. DAPI: DAPI-stained image of hyphae. CFW+DAPI: CFW and DAPI-stained image of hyphae. All scale bars represent 5μm.

Hyphae are the most common resource for preparing protoplast. The morphology of vegetative hyphae is the key factor related to the difficulty of protoplast transformation, such as septate to aseptate and uninucleate to multinucleate. In the genetic engineering of the fungi species with aseptate multinucleate hyphae, heterokaryons are spontaneously formed during transformation. And, it is difficult to screen out the homozygote with engineered nucleus (Kaminskyj and Hamer, 1998). The cell wall of the vegetative hyphae of *C. empetri* MEFC009 was stained by CFW. As shown in Figure 1, the hyphae of *C.empetri* are branched and septate. The length of each cell is about 7–8 μm. The nuclei were stained in bright blue by DAPI. We can observe that the nuclei were equidistantly distributed in hyphae. When stained with DAPI and CFW simultaneously, the nuclei and diaphragm of the hyphae could be observed. It indicated that only one nucleus was present in each septate hyphae cell. This type of vegetative hyphae is the most suitable for genetic transformation.

### Verification of Selectable Markers

A selectable marker is the essential element for genetic transformation, which is usually also a limiting factor for genetic engineering in filamentous fungi. There are a few selectable markers that have been developed in the genetic transformation of filamentous fungi (Ramsden et al., 2011). However, these selectable markers are not always universal in different species because of the different sensitivity to antibiotics. The resistance gene of hygromycin B, *hph*, has been confirmed functional for the transformation of *C. empetri* F-11899 (Yamada et al., 2009). To verify the selectable markers applicable to *C. empetri* MEFC009, the sensitivity to four selectable antibiotics were tested. The growth of *C. empetri* MEFC009 were inhibited on potato dextrose agar (PDA) individually supplemented with 100μg/ml of hygromycin B, geneticin, and nourseothricin, but grew normally on plates supplemented with 500μg/L of pyrithiamine. It demonstrated that *C. empetri* MEFC009 is sensitive to hygromycin B, geneticin, and nourseothricin.

**Figure 2:**
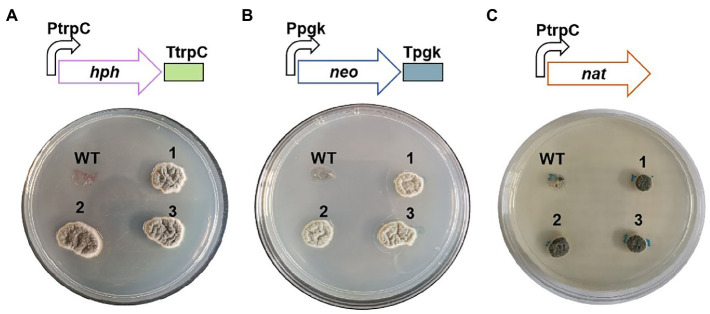
Antibiotics sensitive tests of wild-type *C. empetri* MEFC009 and mutant strains. Mutant strains with resistance genes of *hph* **(A)**, *neo* **(B)**, and *nat* **(C)**. WT: *C. empetri* MEFC009, 1–3: mutant strains.

The plasmids containing the resistant genes of hygromycin B (*hph*), geneticin (*neo*), or nourseothricin (*nat*) were respectively transformed into *C. empetri* MEFC009 using ATMT method as previously described (Yamada et al., 2009). The transformants were successfully screened out on PDA plates containing 100μg/ml of corresponding antibiotics (Figure 2). The results of PCR showed that the resistant genes *hph*, *neo*, and *nat* were integrated into the chromosome of the transformants, respectively, (Supplementary Figure S1). Therefore, the resistant genes *hph*, *neo*, and *nat* are available selectable markers for the transformation of *C. empetri*.

### Development of Protoplast-Mediated Transformation Method

Although the genetic transformation of *C. empetri* MEFC009 was achieved through ATMT method, it is still inefficient, no more than 10 transformants obtained in each experiment. In addition, the transformation process is laborious, time-consuming, and poorly repeatable. Protoplasts-mediated transformation is the most convenient and popular method for the transformation of filamentous fungi (Prabha and Punekar, 2004). The generation of high-quality protoplasts is the major challenge to achieve efficient transformation (Roth and Chilvers, 2019). However, enzymatic digestion conditions of the cell walls are different for each fungal strain. The details should be optimized for each case such as hydrolase composition, isotonic solution, regeneration medium, etc. (Ruiz-Díez, 2002).

**Figure 3:**
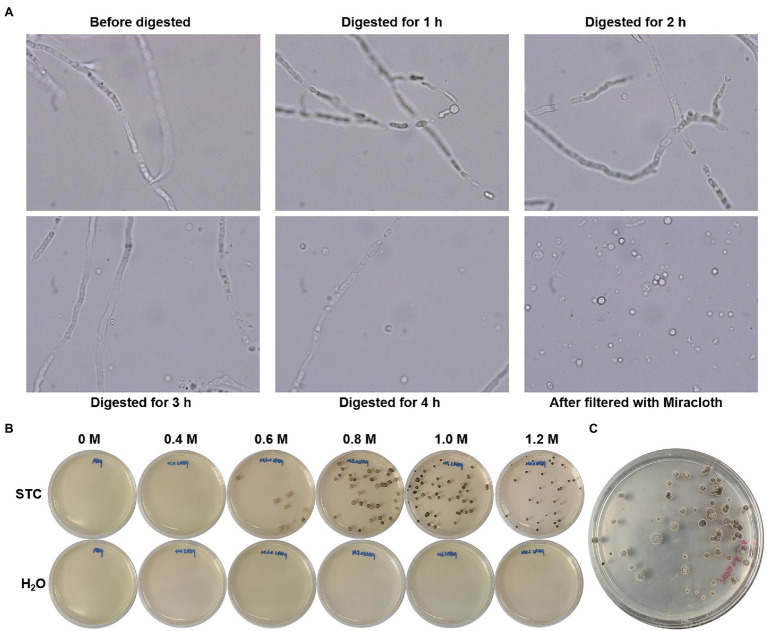
Protoplast-mediated transformation. **(A)** Images of hyphae and protoplasts incubated in the enzyme solution (100×objective lens). **(B)** Optimization of regeneration medium with different concentrations of D-sorbitol; STC: protoplasts were diluted with STC; H_2_O: protoplasts were diluted with H_2_O. **(C)** Original transformation plates of *hph-sgfp*.

We optimized the protoplast transformation according to the previously developed method (Marcone et al., 2010). The hyphae of *C. empetri* MEFC009 were cultivated in PDB medium using a two-step inoculation method. The optimized enzyme solution of 0.6% of snailase, 0.6% lysis enzymes, and 0.7% of lywallzyme was used for cell wall digestion, which produced 1.5×10^6^ protoplasts/ml at 30°C for 2–4h (Figure 3A). The protoplasts were purified and resuspended at 1.5×10^7^ protoplasts/ml in STC solution. In the following PEG-CaCl_2_ mediated transformation, 1μg of DNA fragment of *hph-sgfp* was mixed and incubated with 100μl protoplasts solution. The regeneration medium was optimized, and the protoplasts could regenerate on the PDA plates with 0.8M and 1.0M D-sorbitol (Figure 3B). Beginning with 1.5×10^6^ protoplasts, approximately 100–120 positive transformants were obtained in a reaction using *hph* marker (Figure 3C). The efficiency is significantly higher than that of the ATMT method and is sufficient for functional genomic research and metabolic engineering.

### Evaluation of Promoters

**Figure 4:**
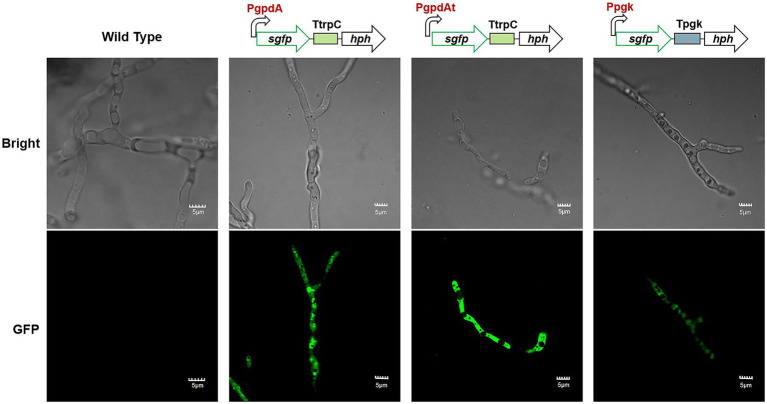
Microscopic features of wild type and representative mutant strains with promoters of *PgpdA*, *PgpdAt*, and *Ppgk*. Bright: Bright-field images. GFP: Fluorescence images. All scale bars represent 5μm.

For a specific fungal host, the efficient promoters are the key elements to achieve high-level expression of target genes (Blumhoff et al., 2013). The promoters of glyceraldehyde-3-phosphate dehydrogenase (*gpd*) and phosphoglycerate kinase (*pgk*) of *A. nidulans* have widely been used in numerous filamentous fungi (Streatfield et al., 1992; Cao et al., 2012). The function of promoters *Ppgk*, *PgpdA* (from *A. nidulans*), and *PgpdAt* (from *A. terreus*; Huang et al., 2014) were evaluated in *C. empetri* MEFC009 using sGFP (synthetic green fluorescent protein) as the reporter. As shown in Figure 4, the DNA cassettes containing the promoters (*PgpdA*, *PgpdAt*, or *Ppgk*), *sgfp*, *TtrpC* terminator, and *hph* were transformed into *C. empetri* MEFC009, respectively. The integration of DNA cassettes in the genome was confirmed by genomic PCR. The hyphae of transformants were randomly picked out and observed under the fluorescence microscope. The transformants harboring *PgpdA*, *PgpdAt*, and *Ppgk* evaluation cassettes displayed significant green fluorescent, while the parental strain did not show any visible fluorescence (Figure 4). These results demonstrated that promoters *PgpdA*, *PgpdAt*, and *Ppgk* successfully drove *sgfp* expression in *C. empetri* MEFC009. According to the intensity of fluorescence, *PgpdA* and *PgpdAt* showed similar promoter activity, which is significantly stronger than that of *Ppgk*.

### Construction of the Chassis Cell With High Gene-Targeting Frequency

**Figure 5:**
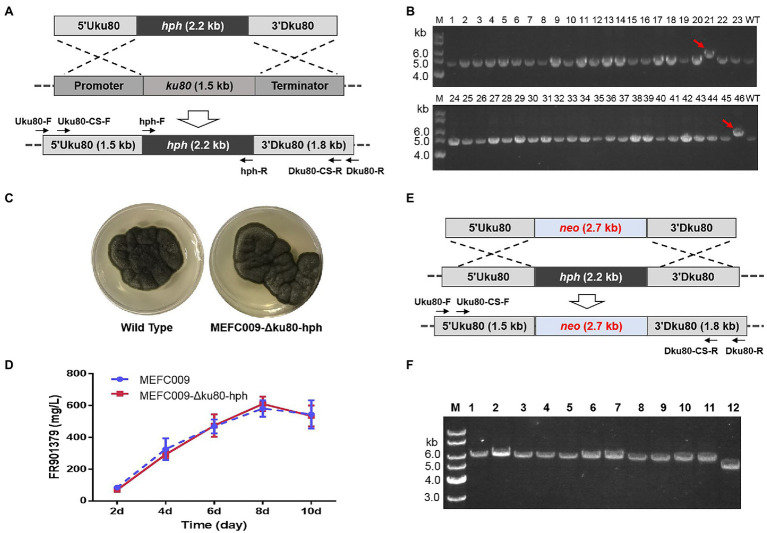
Construction of the chassis cell with high gene-targeting frequency. **(A)** Strategy for deleting *ku80* gene in *C. empetri* MEFC009. **(B)** Genotype verification of transformants using primers Uku80-F/Dku80-R. Lane M: 1kb DNA marker; lane 1–46: transformants; lane WT: *C. empetri* MEFC009. The gene-targeting cassette was integrated at *ku80* loci only in transformants NO.21 and NO.46. **(C)** Comparison of cell growth on PDA plates. **(D)** Comparison of the FR901379 production. **(E)** Strategy for replacing the *hph* marker in *C. empetri* MEFC009-Δku80-hph with *neo*. **(F)** Genotype verification of *C. empetri* MEFC009-Δku80-neo. Lane M: 1kb DNA marker; lane 1–11: transformants; lane 12: parent strain *C. empetri* MEFC009-Δku80-hph.

Gene-targeting is the basis for various genetic engineering techniques, including gene knockout, promoter replacement, and site-specific expression of heterogeneous genes, etc. (Ninomiya et al., 2004). However, homologous recombination (HR) efficiency in filamentous fungi is extremely low because the NHEJ pathway dominates the repair of DNA double-strand breaks (Huang et al., 2016). Learning from previous research, we tried to improve the frequency of gene targeting by disrupting the NHEJ pathway. The *ku80* gene was identified according to the annotation of genome sequencing of *C. empetri* MEFC009. As shown in Figure 5A, 1.5kb region in *ku80* gene was replaced by the *hph* selectable marker. The transformants were screened using PDAS-H plates (PDA with 0.8M D-sorbitol and 100 μg/ml of hygromycin B). Forty-six transformants with hygromycin B resistance were randomly selected for further genotyping. Only in two transformants, the gene-targeting cassette was directly integrated at the *ku80* loci (Figure 5B). Therefore, the frequency of HR was about 4% in *C. empetri*.

The phenotypic characteristics of *C. empetri* MEFC009-Δku80-hph were assessed to evaluate the influences of *ku80* deletion. The *ku80* deletants showed the same growth rates, colonial morphology, and pigmentation on PDA plates (Figure 5C). Furthermore, no deviation in FR901379 productivity was observed in the shake flask fermentation (Figure 5D). To evaluate the HR frequency in the *ku80* deletant, the *hph* integrated in *ku80* loci was replaced with *neo* through another round of gene-targeting (Figure 5E). The transformants were screened by PDAS containing 100μg/ml of geneticin and 11 transformants were randomly selected for genotyping. The results of genomic PCR showed that the *hph* sequence was successfully replaced with *neo* sequence in all 11 transformants (Figure 5F). These results demonstrated that the HR frequency has been improved from 4 to 100% by deleting *ku80* in *C. empetri*.

### Identification of the DHN-Melanin PKS

**Figure 6:**
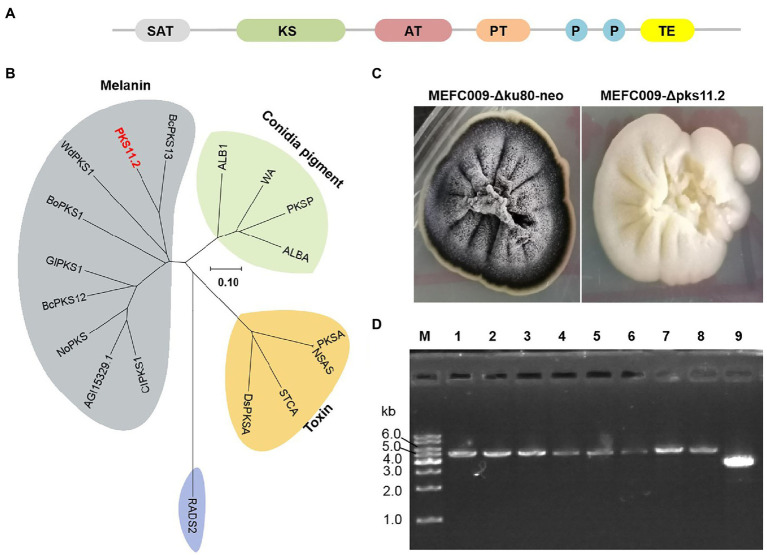
Identification of DHN-melanin PKS in *C. empetri* MEFC009. **(A)** Domain structure of PKS11.2. **(B)** Phylogenetic analysis of PKS11.2. PKS11.2 grouped with the melanin biosynthesis PKSs. The accession numbers of all used PKSs were listed in Supplementary Table S3. **(C)** Colonial morphology of *C. empetri* MEFC009-Δku80-neo and *C. empetri* MEFC009-Δpks11.2. **(D)** Genotype verification of transformants using primers Upks11.2-F/Dpks11.2-R. Lane M: 1kb DNA marker; lane 1–8: *C. empetri* MEFC009-Δpks11.2; lane 9: parent strain *C. empetri* MEFC009-Δku80-neo.

The 1,8-dihydroxynaphthalene (DHN) melanin is the most common type of conidial pigments in ascomycetous fungi, which is polymerized from polyketide naphthopyrone (Eisenman and Casadevall, 2012). The colony of *C. empetri* MEFC009 displays black color on PDA plates. Identifying the synthesis gene of melanin would be a perfect practice for testing the efficiency of genetic modification. According to the results of antiSMASH analysis, there are 18 PKSs in the genome of *C. empetri* MEFC009 (Blin et al., 2019). Among them, PKS11.2 was predicted to be responsible for the synthesis of melanin, which has four potential catalytic domains: beta-ketoacyl synthase (KS), an acyltransferase (AT), two acyl carrier sites (P1 and P2), and a thioesterase (TE)/Claisen cyclase (CYC; Figure 6A). The phylogenetic tree indicates that PKS11.2 appears highly homologous to other identified DHN-melanin PKSs, BcPKS13 of *Botrytis cinerea* (Schumacher, 2016), WdPKS1 of *Wangiella (Exophiala) dermatitidis* (Feng et al., 2001), and BoPKS1 of *Bipolaris oryzae* (Moriwaki et al., 2004; Figure 6B). To identify the function of *pks11.2*, the gene-targeting cassette harboring *hph* selectable marker was transformed in the NHEJ-deficient mutant *C. empetri* MEFC009-Δku80-neo. All the transformants growing on selection plates exhibited white colonies (Supplementary Figure S2; Figure 6C). It indicated that the *pks11.2* was indeed involved in the biosynthesis of melanin and was disrupted by gene-targeting in all transformants. The genotype was further confirmed by genomic PCR, that the selectable marker *hph* was indeed directly integrated at *pks11.2* loci in all eight randomly selected transformants (Figure 6D). Therefore, *C. empetri* MEFC009-Δku80-neo was indeed an excellent chassis cell for gene-targeting.

## Conclusion

*C. empetri* was applied in the industrial production of FR901379, the precursor compound of antifungal agent micafungin. The studies of FR901379 biosynthesis were hampered by the poor genetic transformation system. Given that, we developed an efficient genetic manipulation system in the industrial FR901379-producing strain *C. empetri* MEFC009, including protoplast-mediated transformation method, essential genetic elements of selectable markers and promoters, and chassis cell with high gene-targeting frequency. Based on this genetic manipulation system, we identified the biosynthetic *pks* gene of DHN-melanin in *C. empetri*. This efficient genetic manipulation system will facilitate the research of FR901379 biosynthetic mechanism and further sequential metabolic engineering. And, it is also an indispensable platform for the genome mining of secondary metabolites in *C. empetri*.

## Data Availability Statement

The datasets presented in this study can be found in online repositories. The names of the repository/repositories and accession number(s) can be found in the article/Supplementary Material.

## Author Contributions

XL and XH conceived the project and supervised the research. XH and PM designed the experiments. PM, MW, and JL performed the fungal genetic and fermentation experiments. XL, XH, and PM analyzed all data and wrote the manuscript. All authors contributed to the article and approved the submitted version.

## Conflict of Interest

The authors declare that the research was conducted in the absence of any commercial or financial relationships that could be construed as a potential conflict of interest.

## Publisher’s Note

All claims expressed in this article are solely those of the authors and do not necessarily represent those of their affiliated organizations, or those of the publisher, the editors and the reviewers. Any product that may be evaluated in this article, or claim that may be made by its manufacturer, is not guaranteed or endorsed by the publisher.
