# Supplementary material for: Establishing an Efficient Genetic Manipulation System for Sulfated Echinocandin Producing Fungus Coleophoma empetri
Source: Front Microbiol. 2021 Aug 20;12:734780. doi: 10.3389/fmicb.2021.734780 (PMC8417879; doi:10.3389/fmicb.2021.734780)
Supplement: Supplementary file 1 [file Table_1.DOCX]

**Table S1. Primers used in this study**

| Primers | Sequence (5’→3’) | Purpose |
| --- | --- | --- |
| Ppgk-F | tgcctgcaggtcgactctagagtctagagaaggaaaagcccc | Amplifying the promoter of *Ppgk* |
| Ppgk-R | TGGGTCTTCTCCTTGCCCATtgttgctatagctgtacgga | Amplifying the promoter of *Ppgk* |
| Tpgk-F | ATGCTCGACGAGTTCTTCTAAtaaataaatgaagaattttg | Amplifying the terminator of *Tpgk* |
| Tpgk-R | tacaccaagttaccacaccgattgcagcgcacaagtcagt | Amplifying the terminator of *Tpgk* |
| sgfp-F | atgttgagcaagggcgaggag | Amplifying the gene s*gfp* |
| sgfp-R | cccggccgctttacttgtaca | Amplifying the gene s*gfp* |
| hph-F | ttcgggatcgcaagcgtaaag | Amplifying the gene *hph* |
| hph-R | caattatctttgcgaacccagg | Amplifying the gene *hph* |
| PgpdA-F | ttgatcgagacctaatacagc | Amplifying the cassette of *PgpdA-sgfp-hph* |
| PgpdAt-F | gttacactctgggaggatcc | Amplifying the promoter of *PgpdAt-sgfp-hph* |
| TtrpC-R | ttacctctaaacaagtgtacc | Amplifying the terminator of *PgpdA*/*PgpdAt-sgfp-hph* |
| Uku80-F | tgcgcgtctgaaatggacac | Amplifying the flanking 5’ DNA of gene *ku80* |
| Uku80-(hph)-R | ctttacgcttgcgatcccgaaGGCCATCTGTAACAAGAATAA | Amplifying the flanking 5’ DNA of gene *ku80* |
| Dku80-(hph)-F | ccctgggttcgcaaagataattgCGAGATACAATTACGCCACT | Amplifying the flanking 3’ DNA of gene *ku80* |
| Uku80-(neo)-R | ggggcttttccttctctagaGGCCATCTGTAACAAGAAT | Amplifying the flanking 5’ DNA of gene *ku80* |
| Dku80-(neo)-F | actgacttgtgcgctgcaatCGAGATACAATTACGCCACT | Amplifying the flanking 3’ DNA of gene *ku80* |
| Dku80-R | cctcggaatgtctccagaag | Amplifying the flanking 3’ DNA of gene *ku80* |
| Uku80-CS-F | Tgccattaaggtacctgtgc | Amplifying the cassette of *ku80-hph* |
| Uku80-CS-R | accagatcgacctttagctg | Amplifying the cassette of *ku80-hph* |
| PtrpC-FP | tgcctgcaggtcgactctagagCGACGTTAACTGATATTGAA | Amplifying the gene *nat* |
| nat-RP | tacaccaagttaccacaccgCGACGTTGTAAAACGACGGCC | Amplifying the gene *nat* |
| neo-FP | tccgtacagctatagcaacaATGGGCAAGGAGAAGACCCA | Amplifying the gene *neo* |
| neo-RP | caaaattcttcatttatttaTTAGAAGAACTCGTCGAGCAT | Amplifying the gene *neo* |
| Upks11.2-F | taatgctgatctcgactgtg | Amplifying the flanking 5’ DNA of gene *pks11.2* |
| Upks11.2-R | ctttacgcttgcgatcccgaaGAAGTGTGCTGATGTCTCAA | Amplifying the flanking 5’ DNA of gene *pks11.2* |
| Dpks11.2-F | cctgggttcgcaaagataattgTTTCTATATTTGGGTTTTGC | Amplifying the flanking 3’ DNA of gene *pks11.2* |
| Dpks11.2-R | GTGGGTCGAGCGAGTATCTG | Amplifying the flanking 3’ DNA of gene *pks11.2* |
| Upks11.2-CS-F | ccgtgtcacagcctcttcct | Amplifying the cassette of pks11.2-hph |
| Dpks11.2-CS-R | CTTGAATGCCGTGGGCTTGA | Amplifying the cassette of pks11.2-hph |
