# Supplementary material for: Establishing an Efficient Genetic Manipulation System for Sulfated Echinocandin Producing Fungus Coleophoma empetri
Source: Front Microbiol. 2021 Aug 20;12:734780. doi: 10.3389/fmicb.2021.734780 (PMC8417879; doi:10.3389/fmicb.2021.734780)
Supplement: Supplementary file 2 [file Table_2.DOCX]

**Table S2. Plasmids used in this study**

| Plasmids | Genotypes | Reference of source |
| --- | --- | --- |
| pCambia1300 | Carrying gene of *hph* | (Gu et al., 2018) |
| pPK2natGFPD | Carrying gene of *nat* | (Hršelová et al., 2015) |
| pPM-3 | Carrying gene of *nat* | This study |
| pPM-4 | Carrying gene of *neo* | This study |
| pAN52-4 | Carrying cassette of *PgpdA-sgfp-TtrpC-hph* | Our lab |
| pXH2-1 | Carrying cassette of *PgpdAt-sgfp-TtrpC-hph* | Our lab |
