# Supplementary material for: Establishing an Efficient Genetic Manipulation System for Sulfated Echinocandin Producing Fungus Coleophoma empetri
Source: Front Microbiol. 2021 Aug 20;12:734780. doi: 10.3389/fmicb.2021.734780 (PMC8417879; doi:10.3389/fmicb.2021.734780)
Supplement: Supplementary file 3 [file Table_3.DOCX]

**Table S3 The accession numbers of all used PKSs**

| **Gene ID** | **Gene code** | **Species** |
| --- | --- | --- |
| MZ147973 | PKS11.2 | *Coleophoma empetri* |
| XP_001547095.2 | BcPKS13 | *Botrytis cinerea* |
| AAD31436.3 | WdPKS1 | *Wangiella dermatitidis* |
| BAD22832.1 | BoPKS1 | *Bipolaris oryzae* |
| AAN59953.1 | GlPKS1 | *Glarea lozoyensis* |
| XP_024547375.1 | BcPKS12 | *Botrytis cinerea* |
| AAD38786.1 | NoPKS | *Nodulisporium sp.* |
| BAA18956.1 | ClPKS1 | *Colletotrichum lagenaria* |
| AGI15329.1 | No hit | *Verticillium dahliae* |
| XP_002147717.1 | ALB1 | *Penicillium marneffei* |
| 1905375A | WA | *Aspergillus nidulans* |
| CAA76740.1 | PKSP | *Aspergillus fumigatus* |
| G3XLL5.1 | ALBA | *Aspergillus niger* |
| Q12053.1 | NSAS | *Aspergillus parasiticus* |
| AAR32704.2 | PKSA | *Aspergillus sp.* |
| Q12397.2 | STCA | *Aspergillus nidulans* |
| Q30DW5.1 | DsPKSA | *Dothistroma septosporum* |
| C5H882.1 | RADS2 | *Chaetomium chiversii* |
