# Supplementary material for: Establishing an Efficient Genetic Manipulation System for Sulfated Echinocandin Producing Fungus Coleophoma empetri
Source: Front Microbiol. 2021 Aug 20;12:734780. doi: 10.3389/fmicb.2021.734780 (PMC8417879; doi:10.3389/fmicb.2021.734780)
Supplement: Supplementary file 4 [file Table_4.DOCX]

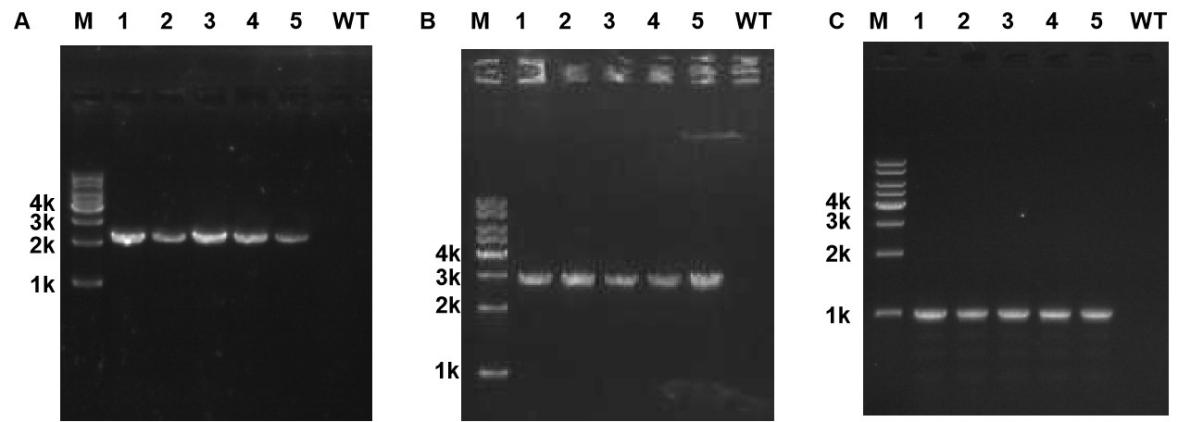


**Figure S1. PCR analysis results of genotypes of transformants.** The transformants were inserted with resistant genes of (A) hygromycin B (*hph*). (B) geneticin (*neo*). (C) or nourseothricin (*nat*). Lane M: 1 kb DNA marker; lane 1-5: transformants; lane WT: Wild-type *C. empetri* MEFC009.


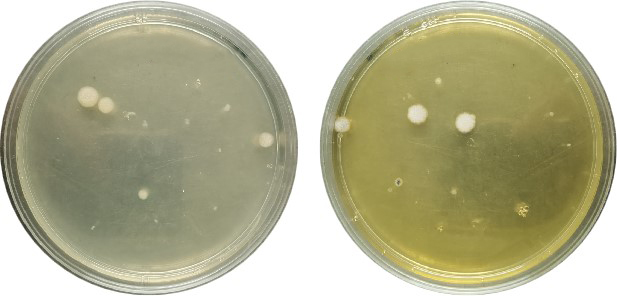


**Figure S2. Original transformation plates of mutants *C. empetri-*∆pks11.2.**
